# Association of anti-PD-(L)1 treatment duration to efficacy in advanced solid tumors: a single center retrospective study

**DOI:** 10.1080/07853890.2025.2476729

**Published:** 2025-03-16

**Authors:** Saara Kuusisalo, Sanna Iivanainen, Jussi P. Koivunen

**Affiliations:** Department of Medical Oncology and Radiotherapy and Medical Research Center Oulu, Oulu University Hospital and University of Oulu, Oulu, Finland

**Keywords:** ICI, anti-PD-(L)1, NSCLC, melanoma, treatment duration, treatment-free survival

## Abstract

**Background:**

Immune checkpoint inhibitors (ICIs) are a standard of care in multiple cancers. Only a minority benefits, thus, optimal use and treatment duration remain indistinct. While biomarkers albeit PD-L1 are scarce, declined performance status and cancer-related systemic inflammation detected by blood inflammatory markers such as C-reactive protein (CRP) have been linked to inferior prognosis.

**Materials and Methods:**

We investigated the association of limited anti-PD-(L)1 treatment duration to therapy efficacy in melanoma and non-small cell lung cancer (NSCLC) patients who received therapy in a non-curative setting in Oulu University Hospital 2014–2022. Baseline prognostic factors (e.g. ECOG, CRP, and PD-L1 for NSCLC) were collected. Progression-free (PFS), overall (OS), and IO-free survival were analyzed using the Kaplan–Meier and Cox regression methods.

**Results:**

126 patients (NSCLC, *n* = 72; melanoma, *n* = 54) were included. Majority (*n* = 101) were treated in the first line. Objective response rate was 34.9%. The median (m) anti-PD-(L)1 treatment duration was 3.42 months (mo). The mPFS and mOS were 6.8 mo (CI 95% 4.4–9.3) and 19.1 mo (CI 95% 13.3–24.9). Of the baseline factors, ECOG and CRP retained their significance in multivariate analysis for PFS (HR 0.34, CI 95% 0.19–0.59; HR 0.34, CI 95% 0.22–054) and OS (HR 0.38, CI 95% 0.20–0.71; HR 0.29, CI 95% 0.17–0.49). No difference was observed in PFS (HR 1.40, CI 95% 0.68–2.90) or OS (HR 0.69, CI 95% 0.29–1.65) according to treatment duration (3-6mo vs. > 6 mo). Long median IO-free survival (10.2 months; CI 95%, 4.1–16.3) was detected.

**Conclusion:**

We characterized an anti-PD-(L)1 treated advanced NSCLC and melanoma cohort in which treatment benefit occurs irrespective of treatment duration and long-term benefit is observed off-treatment.

## Introduction

Immune checkpoint inhibitors (ICIs) have changed the treatment landscape of advanced cancers with long lasting treatment responses even after short therapy courses, as well as improved prognosis in many advanced tumor types. ICIs are approved in multiple cancer types, including melanoma and non-small cell lung cancer (NSCLC) [1–5]. Interestingly, ICI treatments have brought about ongoing treatment responses without a clear association to therapy duration [6–8]. Though ICIs are widely used in cancer care, the optimal treatment duration remains unclear. Typically, in phase III clinical trials of advanced malignancies, ICI treatments have been continued for two years or until disease progression [1–4]. However, data suggest that a more limited duration of therapy can generate comparable treatment results [9–11]. Importantly, while the length of therapy is evaluated in drug regulatory approvals, there are no requirements to determine the optimal duration of therapy when novel treatments are authorized, as long as the positive benefit/risk ratio of the drug is considered established. Consequently, the optimal ICI treatment duration calls for more clinician-initiated research, since more limited treatment duration could potentially decrease ICI-related adverse effects as well as improve patients’ quality of life and provide direct cost savings.

Despite the revolutionary results of ICI therapies in cancer care, only 20%–40% of patients gain treatment benefit. Regardless of extensive research initiatives, there is only a limited number of clinically applicable predictive factors such as tumor PD-L1 staining. Furthermore, the positive and negative predictive values of the identified factors are generally low [12]. Of patient-related prognostic factors, peripheral blood-based biomarkers such as elevated C-reactive protein (CRP) levels have been associated with an inferior survival in anti-PD-(L)1 treated [13,14]. Thus, since biomarkers for treatment benefit are scarce, restraining the treatment duration is an even more important factor in controlling the financial toxicity of ICIs.

According to the local guidance for treating physicians in our clinic, discontinuation of anti-PD-(L)1 treatment after six months of therapy in patients with advanced cancers, even in the absence of disease progression has been a recommended option. However, the therapy may have been continued for a longer treatment interval based on physicians’ clinical judgement surpassing the advised time frame of six months. Therefore, our patient material provides a unique cohort to investigate the effects of variable treatment durations and the grounds of clinical decision making of therapy efficacy in association to treatment length. Since good quality randomized evidence for the optimal anti-PD-(L)1 treatment duration is limited, our cohort can provide valuable insights into this clinically meaningful and controversial issue.

In the current work, we investigated the association of anti-PD-(L)1 treatment duration to treatment outcomes in advanced melanoma and NSCLC patients. We focused the analysis on these two tumor types since ICI indications for these disease entities have the longest follow-up data available, and to elucidate the effects of anti-PD-(L)1 therapy irrespectively of indication. Our main hypothesis was that a limited duration of anti-PD-(L)1 treatment would produce equally effective long-term treatment results when compared to registration trials.

## Materials and methods

Cancer patients with melanoma or NSCLC treated in Oulu University Hospital during 2014-2022 were retrospectively identified from the pharmacy or pathology records. The inclusion criteria were NSCLC or melanoma diagnosis, at least one dose of anti-PD-(L)1 therapy as a single or combination therapy, and complete treatment history available in the electronic patient records. For NSCLC patients, the PD-L1 tumor proportional score (TPS) staining result was required as an inclusion criterion. The exclusion criteria were other malignancy than NSCLC or melanoma, no anti-PD-(L)1 therapy received, or anti-PD-(L)1 therapy received in adjuvant settings. All the cases were manually reviewed and patients not meeting the inclusion or fulfilling exclusion criteria were removed based on the judgment of the principal investigators (SI and JK).

Data collected from the electronic patient records included date of birth, gender, ECOG score at the time of non-curative stage, cancer type, date of diagnosis, routine laboratory measures including CRP, treatment line, the first and last date of anti-PD-(L)1 containing therapy, best overall response (BOR), reason for therapy discontinuation, date of tumor progression on anti-PD-(L)1 containing therapy, or the last day of follow-up, re-challenge of anti-PD-(L)1 therapy, BOR of re-challenge, and date of death, or the last day of follow-up. For CRP, a cut-off of 10 mg/ml was applied, based on previous publications [14].

Overall survival (OS) was calculated from the date of the first anti-PD-(L)1 infusion to the date of death or end of follow-up, the first counted as an event. Progression-free survival (PFS) was calculated from the first date of anti-PD-(L)1 infusion to the documented tumor progression, death, or end of follow-up, while tumor progression and/or death were counted as an event. IO free-survival was calculated from the last date of anti-PD-(L)1 infusion to the next active treatment initiation date or end of follow-up, the first counted as an event. Patients whose next event was death and/or initiation of best supportive care were excluded from IO-free survival analysis to focus on patients who were eligible for further therapies. IBM SPSS Statistics 27.00.00 for Windows was applied for statistical analysis. Survival was analyzed using the Kaplan–Meier and Cox regression methods with 95% confidence intervals.

Data collection was carried out according to national legislation and under a permit from the medical director of Oulu University Hospital (study no. 299/2016). Pseudonymization was carried out before data analysis. Due to the national legislation and the retrospective nature of the study, the ethics committee of Northern Osthrobothnia Hospital District waived the need for ethical approval. Informed consent was not sought due to the registry nature of the study.

## Results

### Patient cohort

A total of 126 patients with NSCLC (*n* = 72) and melanoma (*n* = 54) treated with anti-PD-(L)1 based therapies in a non-curative setting were included in the study. None of the included NSCLC patients bared targetable genetic tumor alterations (EGFR, ALK, or ROS1). Patient demographics are presented in detail in Table 1. In brief, most of the patients received anti-PD-(L)1 therapy in first-line settings (*n* = 101, 80.2%). The majority of the patients received single-anti-PD-(L)1 therapy (*n* = 114, 90.5%) whereas the rest were treated with combination therapy mostly with histology matched chemotherapy for NSCLC (Keynote 189, Keynote 407). Objective response rate (ORR) was 34.9% in the whole cohort. The median anti-PD-(L)1 treatment duration was 3.42 months. Nearly half of the patients (48.4%, *n* = 61) received the therapy under three months, 32.5% three to six months (*n* = 41), and 19% (*n* = 24) over six months. ECOG performance status was available for 116 patients, of whom 99 (78.6%) had ECOG 0-1 and the remaining 17 (13.5%) ECOG 2 or more.

**Table 1.** Patient demographics.

	*n* (%)
N	126 (100)
Age (median), years	68.0
Sex	
Male	94 (74.6)
Female	32 (25.4)
Cancer type	
Non-small cell lung cancer	72 (57.1)
Melanoma	54 (42.9)
ECOG	
Total	116 (92.1)
0–1	99 (78.6)
2 or more	17 (13.5)
ICI-treatment line	
1st	101 (80.2)
2nd or later	25 (19.8)
ICI therapies	
Pembrolizumab	87 (69.0)
Nivolumab	32 (25.4)
Durvalumab	7 (5.6)
Treatment duration (median), months	3.42
Treatment duration	
< 3 months	61 (48.4)
3–6 months	41 (32.5)
> 6 months	24 (19)
Reason for ICI-treatment discontinuation	
Total	118 (93.7)
PD	60 (47.6)
Adverse event	25 (19.8)
Planned duration fulfilled	32 (25.4)
CR	1 (0.8)
Best overall response of ICI therapy	
CR	12 (9.5)
PR	32 (25.4)
SD	25 (19.8)
PD	47 (37.3)
NA	10 (7.9)

Values are presented as *n* (%) unless indicated otherwise. ICI: Immune Checkpoint Inhibitor; PD: progressive disease; CR: complete response; PR: partial response; SD: stable disease; NA: not applicable.

### Survival analysis

**Figure 1.**
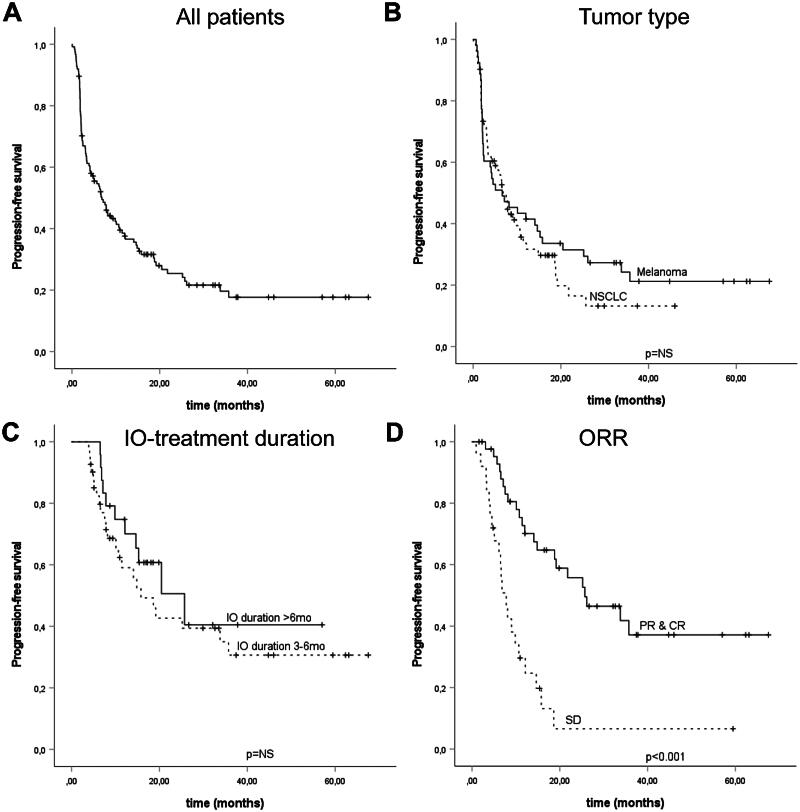
Kaplan–Meir Analysis for progression-free survival in the whole cohort (A), and stratified according to tumor type (B), treatment duration (C), and by treatment response (D). Crosses indicate censored events.

First, we analyzed the cohort for PFS. The median PFS in the cohort was 6.8 months (CI 95% 4.4–9.3). In NSCLC cohort, the mPFS was 6.8 months (CI 95% 5.0–8.6) while the mPFS of 6.6 months was reached in melanoma patients (CI 95% 0.5–12.8). Of the baseline factors, only ECOG score (0–1 vs. 2; HR 0.35, CI 95% 0.20–0.60) and CRP level (≤10 vs. >10; HR 0.34, CI 95% 0.22–0.54) were associated with an improved PFS. PD-L1 TPS was left out of the final analysis since this was available only for the NSCLC patients (TPS ≥50% vs. < 50%, HR 0.62, CI 95% 0.34–1.12). Furthermore, both ECOG score (HR 0.34, CI 95% 0.19–0.59) and CRP (HR 0.34, CI 95% 0.22–0.54) retained their significance in the multivariate analysis (Table 2). In the Kaplan-Meier analysis, ORR was associated with improved PFS (*p* < .001) (Figure 1).

**Table 2.** Univariate and multivariate analysis for PFS.

	Univariate		Multivariate	
	HR	CI (95%)	HR	CI (95%)
Gender				
Female vs. Male	0.712	0.437–1.161		
Tumor type				
Melanoma vs. NSCLC	0.847	0.555–1.293		
ECOG				
0–1 vs. 2	0.348	0.203–0.596	0.336	0.192–0.588
Age				
<70 y vs. ≥ 70 y	0.859	0.566–1.304		
CRP				
≤10 vs. >10	0.344	0.218–0.543	0.342	0.216–0.544
Treatment line				
1st vs later	0.722	0.439–1.186		

**Figure 2.**
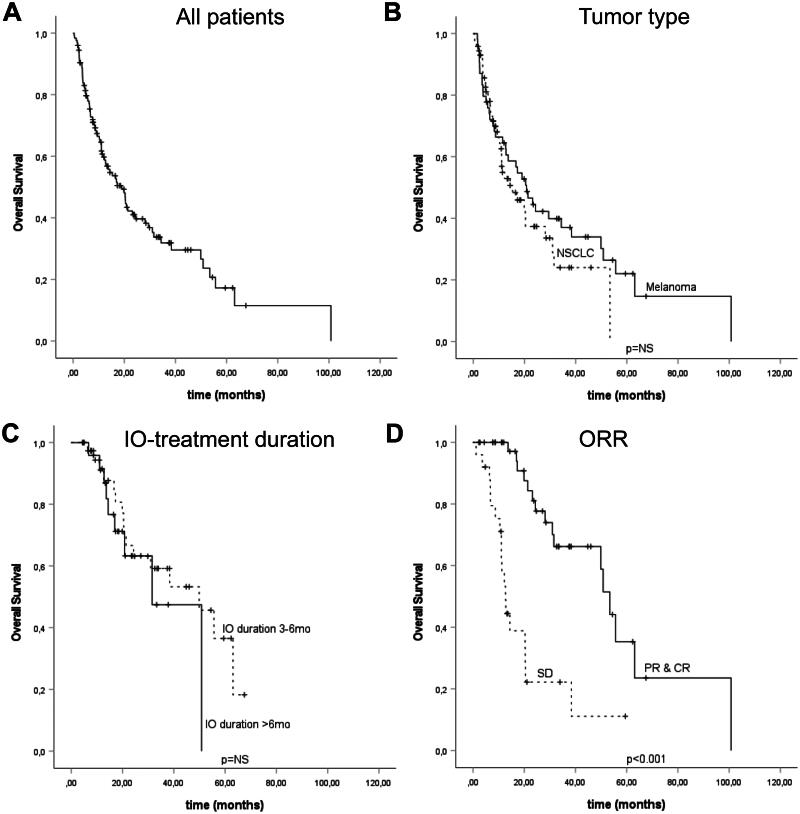
Kaplan–Meir Analysis for overall survival in the whole cohort (A), and stratified according to tumor type (B), treatment duration (C), and by treatment response (D). Crosses indicate censored events.

In the overall survival analysis, the median OS for the whole cohort was 19.1 months (CI 95% 13.3–24.9), 15.4 months (CI 95% 7.0–23.8) in NSCLCs and 20.8 months (CI 95% 13.6–28.0) in the melanoma cohort. In the univariate analysis for OS, ECOG score (HR 0.36, CI 95% 0.20–0.67) and CRP (≤10 vs. >10; HR 0.34, CI 95% 0.22–0.54) were the only factors associated with improved survival while no association was seen with other baseline factors such as tumor type or treatment line (Table 3). PD-L1 TPS was left out of the final analysis since this was available only for the NSCLC patients (TPS ≥50% vs. < 50%, HR 0.53, CI 95% 0.27–1.04). Both ECOG score (HR 0.38, CI 95% 0.20–0.71) and CRP (HR 0.29, CI 95% 0.17–0.49) retained their significance in the multivariate analysis (Table 3). In the Kaplan-Meier analysis, ORR was associated with improved OS (*p* < .001) (Figure 2).

**Table 3.** Univariate and multivariate analysis for OS.

	Univariate		Multivariate	
	HR	CI (95%)	HR	CI (95%)
Gender				
Female vs. Male	0.606	0.343–1.070		
Tumor type				
Melanoma vs. NSCLC	0.782	0.488–1.255		
ECOG				
0–1 vs. 2	0.363	0.199–0.661	0.380	0.203–0.709
Age				
<70y vs. ≥ 70 y	0.982	0.615–1.567		
CRP				
≤10 vs. >10	0.344	0.218–0.543	0.291	0.174–0.487
Treatment line				
1st vs later	0.751	0.445–1.269		

### Treatment duration and its relation to survival

Next, we sought to analyze whether treatment duration is associated with survival. Since we did not observe any survival difference based on tumor type or treatment line, further analysis of treatment duration was considered feasible using the whole cohort. We focused our analysis on patients with a treatment duration of over three months since most of the patients with a shorter treatment length had progressive disease (PD) as BOR (*n* = 44, 72.1%) and a very poor mPFS (2.0 months, CI 95% 1.9–2.2) and mOS (6.4 months, CI 95% 4.1–8.8). In our cohort, 41 (32.5%) patients had a treatment duration of 3-6 months while 24 (19%) patients had a longer treatment duration (Table 1). We did not observe any difference in PFS (HR 1.40, CI 95% 0.68–2.90) or OS (HR 0.69, CI 95% 0.29–1.65) according to treatment duration (3–6 months vs. > 6 months) (Figures 1C and 2C). Since the prognosis of patients with SD compared to PR/CR differed, we performed a further analysis of PFS and OS according to BOR. We did not detect any difference in PFS or OS outcomes stratified by overall response (SD vs. PR/CR) and treatment duration (3–6 months vs. >6 months) (Supplementary Table 1).

### IO-free survival

To study the clinical benefit of ICI therapies surpassing the treatment period, we analyzed the cohort for IO-free survival. IO-free survival was determined as the time from the last dose of ICI therapy to the initiation of the next treatment, or end of follow-up. At the end of follow-up, 118 patients of 126 had had the anti-PD-(L)1 therapy regimen discontinued, the majority of them (*n* = 60, 47.6%) due to PD. Adverse events were the reason of discontinuation for 25 patients (19.8%) while with 32 patients (25.4%), the planned therapy duration had been reached. For one patient, complete response had been the reason for therapy discontinuation. We excluded patients whose next treatment was best supportive care or death to restrict the analysis on patients eligible for further active treatments. This resulted in a cohort of 72 patients, of which 41 (56.9%) had a next line treatment initiated while the rest (*n* = 31, 43.1%) had had no further treatment at the end of follow-up. The cohort included 49 (68.1%) NSCLC and 23 (31.9%) melanoma patients. Among the IO-free survival patients, initial ORR was 51.4%.

**Figure 3.**
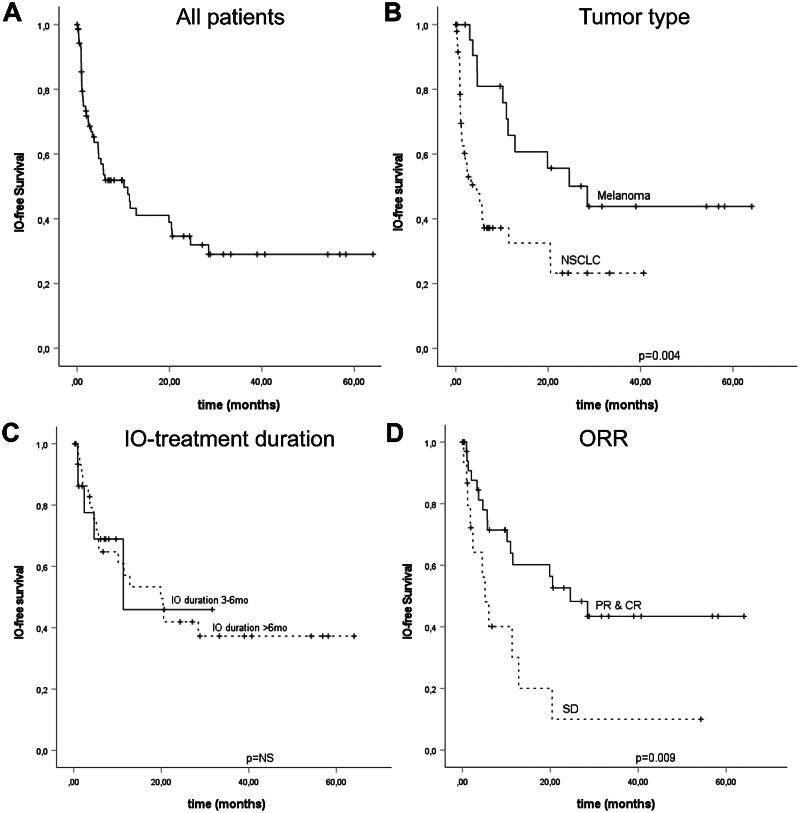
Kaplan–Meir Analysis for IO-free survival in the whole cohort (A), and stratified according to tumor type (B), treatment duration (C), and by treatment response (D). Crosses indicate censored events.

In the analysis, median IO-free survival was 10.2 months (CI 95%, 4.1–16.3). At 24 months, IO-free survival was 32%. (Figure 3A). A significantly longer IO-free survival was seen with melanoma patients compared to NSCLCs (*p* = .004) and in patients with complete response (CR) and partial response (PR) compared to stable disease (SD) (*p* = .009) (Figure 3B,D). However, no difference in IO-free survival was observed according to the treatment duration (3–6 vs > 6 months) (Figure 3C).

Next, we carried out an analysis of IO free survival at specific time points, corresponding responses and anti-PD-(L)1 re-initiation according to response. We did not observe any anti-PD-(L)1 re-initiations in patient completing IO free at 36 months. At earlier time points, IO re-initiations were seen and most of them occurred at within the first 12 months of IO stoppage. According to RECIST 1.1 responses, none of the CR patients required IO therapy re-initiation (Supplementary Table 2).

### ICI re-challenge

Of the 41 patients who had had therapy re-initiated after the first anti-PD-(L)1 containing therapy break, 13 patients were re-challenged with the same drug class as the next line of therapy. Of the re-challenge patients, ten patients (76.9%) had PR as BOR of the first ICI therapy course while the rest had SD (23.1%). Median PFS was 15.8 months and IO-free survival was 11.5 months in the whole cohort. As a result of the anti-PD-(L)1 re-challenge, two (15.4%) patients had PR, five (38.5%) SD, and six (46.2%) PD as BOR, respectively. Median PFS for the re-challenged was 9.7 months (Table 4).

**Table 4.** ICI re-challenge.

	*n* (%)
N	13 (100)
First ICI treatment response	
CR	0 (0)
PR	10 (76.9)
SD	3 (23.1)
PD	0 (0)
Cancer type	
Non-small cell lung cancer	5 (38.5)
Melanoma	8 (61.5)
PFS on the first ICI course (median), months	15.8
IO-free survival (median), months	11.5
ICI re-challenge response	
CR	0 (0)
PR	2 (15.4)
SD	5 (38.5)
PD	6 (46.2)
PFS on ICI re-challenge (median)	9.7

Values are presented as *n* (%) unless indicated otherwise. CR: complete response; PR: partial response; SD: stable disease; PD: progressive disease; PFS: progression-free survival.

## Discussion

In this study, we characterized an anti-PD-(L)1 treated advanced NSCLC and melanoma real-world cohort among whom a limited duration of therapy was applied in the absence of disease progression. The aims of the study were to analyze anti-PD-(L)1 therapy duration and its relation to treatment outcomes, therapy-free survival after treatment discontinuation, and responses to anti-PD-(L)1 therapy regimen re-challenge. We observed no relationship between the therapy duration and survival. Instead, a long therapy-free survival was observed after anti-PD-(L)1 discontinuation while inferior ORR and shorter PFS compared to the initial therapy course was seen among the anti-PD(L)1 re-challenged patients.

The optimal therapy duration with anti-PD-(L)1 treatments remains debatable. Typically, registration trials have investigated a treatment length until disease progression or up to one or two years of therapy, in the absence of obvious clinical or pharmacological rationale. Interestingly, single-agent anti-CTLA-4 treatments were originally approved as a four-cycle regimen (Q3W) without any maintenance treatment, suggesting that the treatment benefit of immune checkpoint therapy might not require a continuous treatment [6]. Currently, there is only one published randomized controlled trial (RCT) investigating a limited treatment duration of anti-PD-(L)1 therapy [15]. In this trial, advanced NSCLC patients receiving nivolumab as a second line therapy were randomized 1:1 to receive continuous treatment or discontinue the therapy after one year. Even though PFS and OS benefits were observed in the continuous treatment group, when interpreting the results, it should be noted that the trial did not stratify patients according to histology, PD-L1 score, or treatment response, thus, in the fixed treatment duration group there were less patients with PD-L1 TPS ≥50%, and more RECIST 1.1 PDs at the time of randomization. Therefore, it is possible that more patients with adverse prognosis and inferior predictive factors were randomized to the fixed treatment arm, and this might play a role in the observed results. Imbalances in terms of prognostic and predictive factors may, obviously be present in our cohort as well, but notably, the treatment duration in our data set was primarily determined based on clinical judgment independently of these factors. Interestingly, superb treatment outcomes in the neo-adjuvant immunotherapy context of NSCLC, melanoma, and MSI high rectal cancer also suggest that even a short course of therapy (4-9 weeks) can lead to durable pathological responses, event-free survival, and overall survival [16–18].

Our study adds knowledge to the field of anti-PD-1 therapy patient selection and efficacy evaluation in a real-world context. The results suggest that continuing the treatment over six months may not improve treatment outcomes. When matching our RWE on therapy efficacy to the trials with comparable patient cohorts, similar median PFS of 6.8 and OS of 15.4 months can be seen in NSCLC patients (KEYNOTE-042: pembrolizumab mPFS 5.6 mo, mOS 16.8 mo) while a decline in survival in melanoma cohort is notable (median PFS of 6.6 and OS of 20.8 months; KEYNOTE-006: pembrolizumab mPFS 8.6 mo, mOS 32.7 mo) [2,5]. Reason for the survival gap seen in RWD of melanoma patients of our cohort is unclear, one possible explanation could be the low number of patients receiving the preferred standard of combination immunotherapy instead of single-anti-PD-(L)1 treatment. However, also in other previous studies, real-world outcomes of anti-PD-(L)1 therapies have typically been inferior to registrational trials, thereby supporting the external validity of our findings [19].

In the current study, we provided a new time-to-event efficacy parameter, IO-free survival, a measure of how long patients could remain free of additional cancer therapies. We observed a long median IO-free survival (10.2 months) in the whole population, and Kaplan–Meier curves plateaued at ∼30%, while no association to treatment duration was observed. This finding further supports the theory that anti-PD-(L)1 treatment benefit occurs irrespectively of treatment duration, and long-term effect is not bound to a continuous treatment. The observed results were irrespective of tumor type, and, thus, may be attributed to the drug class effect.

There are few studies on anti-PD-(L)1 therapy re-challenge in the RWE context. In our cohort, ORR for re-challenge was 15.4% while higher rates have been reported in some of the previous studies [20]. However, re-challenge studies have exclusively focused on patients whose ICI therapy was discontinued due to immune-related adverse events (only ∼20% in our cohort) and, therefore they may not represent similar populations. Even though re-challenge ORR was low in our cohort, to us this does not suggest that the shorter anti-PD-(L)1 therapy would be inferior. Of note, therapy re-initiation was not required for ∼30% of the patients. A long median IO-free survival was observed in the whole cohort while the effect was more sustained among the patients with PR/CR response. This is in line with previous studies which have reported an improved prognosis of patients with CR or PR reponse of ICIs [21,22]. In addition, it should be noted that 38.5% of the re-challenged patients had a SD as a BOR, even though having PD at the initiation of the anti-PD-(L)1 re-challenge.

The development of acquired resistance to ICIs is somewhat poorly defined and lacks throughout biological understanding [23,24]. Even though it is a common clinical phenomenon, the rates of acquired resistance are relatively incompletely reported in literature, and the methodology to identify such patients is inadequately characterized. Furthermore, the level of continuous drug exposure that is required at the time of acquired resistance to ICIs, remains unknown. Previous data indicate that patients can have ongoing responses after discontinuation of ICIs which is uncommon with other molecularly targeted therapies. This suggests that the development of acquired resistance to ICIs might occur irrespectively of whether the patient is on or off therapy [25–27]. Our data, indicating that a short, fixed treatment duration may provide durable responses, and similar survival compared to treatment until disease progression, and that the drug effect and ICI sensitivity may be retained at least in part, indirectly supports this hypothesis.

Our study has some obvious limitations. The study design is retrospective, and this generates apparent uncertainty to the results. The size of the cohort is limited for the subgroup analysis of IO-free survival or therapeutic regimen (single or combination) and multivariate analysis could not be performed in these subgroups. We focused the analysis to NSCLC and melanoma since these indications have the most comprehensive data of anti-PD-(L)1 therapies (due to early approval years and first line indication) and, therefore, the results might not be generalizable to other tumor types. In addition, our cohort is exclusively of Caucasian ethnicity and validity to other ethnicities is questionable.

## Conclusion

In conclusion, we present RWE of a cohort of anti-PD-(L)1 treated NSCLCs and melanomas with fixed treatment duration. The study showed that treatment benefit occurs irrespectively of treatment duration, when three-, and six-months cut-offs are applied, and a long-term therapeutic benefit was observed off-treatment. The study strongly supports the evaluation of limited length anti-PD-(L)1 therapy duration to treatment outcomes in a randomized setting.

## Supplementary Material

Supplemental Material

## Data Availability

The datasets generated and/or analyzed during the current study are not publicly available but are available from the corresponding author on reasonable request.
