# Supplementary material for: Association of anti-PD-(L)1 treatment duration to efficacy in advanced solid tumors: a single center retrospective study
Source: Ann Med. 2025 Mar 16;57(1):2476729. doi: 10.1080/07853890.2025.2476729 (PMC11915729; doi:10.1080/07853890.2025.2476729)
Supplement: Supplemental Material [file IANN_A_2476729_SM8678.docx]

**Supplementary Table 1.** Univariate analysis according to tumor response and treatment duration

|  | **Univariate** | |
| --- | --- | --- |
|  | **HR** | **CI (95%)** |
| **PFS** |  |  |
| SD |  |  |
| 3-6 vs. >6 months | 1.246 | 0.399-3.885 |
| PR/CR |  |  |
| 3-6 vs. >6 months | 0.285 | 0.065-1.260 |
| **OS** |  |  |
| SD |  |  |
| 3-6 vs. >6 months | 1.103 | 0.300-4.055 |
| PR/CR |  |  |
| 3-6 vs. >6 months | 2.183 | 0.609-7.826 |

**Supplementary Table 2.** IO therapy re-initiation and RECIST 1.1 defined responses

|  | **n** | **IO re-initiation n (%)** |
| --- | --- | --- |
| **IO free survival** |  |  |
| 48kk | 4 (100) | 0 (0) |
| RECIST 1.1 |  |  |
| CR | 1 (25) | 0 (0) |
| PR | 2 (50) | 0 (0) |
| SD | 1 (25) | 0 (0) |
| NE | 0 (0) | 0 (0) |
| 36kk | 6 (100) | 0 (0) |
| RECIST 1.1 |  |  |
| CR | 3 (50) | 0 (0) |
| PR | 2 (33.3) | 0 (0) |
| SD | 1 (16.7) | 0 (0) |
| NE | 0 (0) | 0 (0) |
| 24kk | 16 (100) | 2 (12.5) |
| RECIST 1.1 |  |  |
| CR | 5 (31.3) | 0 (0) |
| PR | 9 (56.3) | 2 (100) |
| SD | 1 (6.3) | 0 (0) |
| NE | 1 (6.3) | 0 (0) |
| 12kk | 23 (100) | 6 (26.1) |
| RECIST 1.1 |  |  |
| CR | 6 (26.1) | 0 (0) |
| PR | 12 (52.2) | 4 (66.7) |
| SD | 4 (17.4) | 2 (33.3) |
| NE | 1 (4.3) | 0 (0) |
| 6kk | 35 (100) | 9 (25.7) |
| RECIST 1.1 |  |  |
| CR | 7 (20) | 0 (0) |
| PR | 17 (48.6) | 6 (66.7) |
| SD | 7 (20) | 3 (33.3) |
| NE | 4 (11.4) | 0 (0) |
